# Distributed Diagnoses Based on Constructing a Private Chain via a Public Network

**DOI:** 10.3390/e25091305

**Published:** 2023-09-07

**Authors:** Bing Li, Choujun Zhan

**Affiliations:** 1 School of Economics, Wuhan University of Technology, Wuhan 430070, China; 2 School of Computer, South China Normal University, Guangzhou 510631, China

**Keywords:** distributed diagnoses, secure consultation, medical technology

## Abstract

Secure online consultations can provide convenient medical services to patients who require experts from different regions. Moreover, this process can save time, which is critical in emergency cases, and cut medical costs. However, medical services need a high level of privacy protection that advances the difficulty of a construction method. It is a good idea to construct a virtual private chain through public networks by means of cryptology and identity verification. For this purpose, novel protocols are proposed to finish the package layout, secure transmission, and authorization. By mining the special characteristics of this application, two different kinds of encryption channels were designed to support the proposed protocol to ensure the secure transmission of data. And Hash values and multiple checking were employed in the transmission package to find the incompleteness of data related to network errors or attacks. Besides the secure communication of medical information, the Extended Chinese Remainder Theorem was utilized to finish the approval during a change in committee in emergency situations. Finally, example case was used to verify the effectiveness of the total methods.

## 1. Introduction

It is critical to find novel methods to provide secure and convenient medical consultations during the spread of infectious diseases, such as COVID-19. In this work, a secure scheme used to organize online medical consultation conferences is developed without a large input of time and money with the help of computer technology, including a complete communication protocol, encryption design, and the authorization of private documents. An online medical conference can provide long-distance medical consultations to help patients who need experts in different medical domains or lack the ability to attend specialized hospitals. In addition, this method may cut medical costs, which will enable more people to obtain medical care. Moreover, it is very useful in terms of protecting the health of doctors and patients in epidemic situations. 

Intelligent medical methods are a current trend that combine medical treatment with computer technology or the Internet of Things [1,2,3]. There are several main directions in the development of current technology, including big data analysis [4] and medical image research [5,6]. This work is mainly concerned with intelligent communication processes. The Internet is the most convenient and rapid communication method due to the development of hardware devices and software technology. Hardware devices, especially intelligent equipment, improve the effectiveness of communication [7]. However, the common methods used by general communication networks are not suitable in some special cases, such as underwater applications [8]. These methods do not use universal information exchange; thus, special domains need additional research and inputs to satisfy their specific requirements. In general, there are two ways to meet these special demands. One way is to develop novel information transmission methods based on expert hardware devices. For example, the optimization of supermirrors enables the advanced collection efficiency of X-rays, the signal-to-noise ratio of which reaches 71.8 dB at 100 km and 50.1 dB at 1000 km [9]. An axial grid ionization system was designed and a neutron irradiator was tested to assess the performance [10]. This type of method obviously produces a large improvement in performance compared to that of current systems. However, this approach requires additional hardware devices with large inputs of money and time. Thus, the schema are not always suitable for all situations. With the help of available systems, the second method constructs a high-level protocol that can satisfy the requirements of special applications. Stress management always needs cognitive behavioral treatment, with the doctor and patient being based in the same location. Moreover, general meetings between the doctor and patients will reinforce the effectiveness of treatment [11,12,13]. The Internet enables these treatments to be provided without the limitations of time and place [13].

Besides those topics introduced in the last paragraph, another important research direction involves how to transmit data in a secure format through the Internet [14,15]. An authentication scheme based on cryptographic theory has the ability to securely communicate health data through public networks. However, pre-communication before the normal process is generally necessary, which intensifies the difficulty in special domains [16]. In order to evade this requirement, offering secure access to medical data in an encrypted format for special purposes has been proposed [17], which is useful in terms of transmitting the data through an online model. However, the scope of this research is sufficiently restrictive that the construction of a general communication cannot take place. A three-factor authentication protocol was proposed to support data security and modification in smart medical systems [18]. However, this process always needs stringent verification of biometric identity, which reduces its applicability. Many methods in this catalogue depend on the development of intelligent medical technology [19,20,21]; thus, new technology related to this topic is always being discussed. A novel communication scheme was designed to defend systems against privacy attacks, with the employment of encryption functions required to enhance their robustness and performance [22]. 

Based on the above discussion, it can be seen that the modification of current public communication protocols is a quick and energy-conserving method used to realize online medical consultation conferences between patients and doctors in different hospitals. Of course, hardware devices can enhance the improvement in effectiveness, such as building special communication channels for medical data. However, this approach also means that the period of construction and input of money will dramatically increase. To ensure feasibility, we should build a prototype to verify the effectiveness of this idea, as the development of computer technology can provide sufficient support to modify current communication protocols to satisfy the requirements of online medical consultation conferences. In this context, the security of data and authorizations must be solved. Previous studies have developed some schemes to promote this topic from different standpoints. A complete method, which can maintain the balance between requirements (security and completeness of data) and additional inputs (cost and time), is still lacking. In this work, a complete plan, including the authorization of identity and secure transmission of medical records, is discussed. In short, the main topics are summarized as follows. 

(1) A Special Medical Encryption Dataflow (SMED) is proposed, which will provide a complete framework to organize online medical consultation conferences. The relationships between four roles (namely the patient, doctor, hospital, and Online Medical Center) are defined to solve authorization and permission setting. SMED was designed based on the analysis of the uneven frequency of roles, which balances the security and computational cost by reducing the casual distribution of keys. 

(2) The Secure Medical Image and Character Transmission Protocol (SMICTP) describes the flow frame of medical record transmission, which uses a double-coding system to improve the recognition of transmission errors. Shift and Inverse Shift Functions are defined to achieve this goal. Moreover, the length of data is recorded to provide simple verification with little additional cost. 

(3) Complete and Safe Medical Package (CSMP) and Secure Protocol of Medical Online Conferences (SPMOC) solve the technology problems in SMICTP and SMED. CSMP describes the coding and decoding of the package in a secure way to finish the communication through public networks. In particular, Hash values are input into the format of data according to CSMP, which can ensure that modifications occurring in network attacks can be found. SPMOC elaborates two different types of security channels. Firstly, Channel-HT employs the theory of symmetrical encryption to enable the secure communication of materials. Then, Channel-DT focuses on the approval of unpacking medical records defined by Con-key. The correctness of Channel-DT is verified via the proposed Extended Chinese Remainder Theorem, which has been proven to effectively solve the uncertain approval. 

As a result, the contributions of this work can be summarized as follows:

(1) The secure and reliable framework to organize online medical conferences produces a novel model used to hold doctor consultations, which is helpful in cutting medical costs; thus, more people can obtain medical care in special situations, such as those involving geographical barriers or epidemics.

(2) The complete transmission protocol, including the design of packages and encryption of data in public networks, uses existing hardware to communicate while protecting the private information of patients. Reusing existing hardware facilitates medical online conferences without requiring large additional inputs. 

(3) The Extended Chinese Remainder Theorem relaxes the conditions of authorization to unpack the medical materials, which is more suitable for real-world applications. 

The rest of work is organized as follows: Section 2 presents the basic theory to help understand the work in this study; Section 3 introduces the complete method, with a discussion of the completeness and security of the data; Section 4 presents a case study to show the application of the proposed theory; Section 5 is the conclusion. 

## 2. Background

In this section, some of the basic theory is introduced, which is useful in terms of understanding the methods proposed in Section 3. Two parts of the basic theory are included. Firstly, the Hash function is used to verify the completeness of the data, which ensures that the data are not modified during the communication process through the Internet. The other aspect is the encryption technology of symmetric keys.

### 2.1. Hash Function

The basic purpose of the Hash function [23,24] is to transmit the data with an information abstract, which is a calculated result of the function. When the data package arrives at the receiver, the information abstract is checked against the new result calculated via the receiver according to the before appoints of the communication participants. Since the result provided by the Hash function is not inverse, the two equal information abstracts mean that no changes occur in the transmission. In order to make this point clear, the mathematical expression used is shown below.

**Definition** **1.** *A Hash system is $HIF=\left\{O,Z,K,HF\right\}$ [23,24], where O is the set of data, Z is the set of information, K is the set of keys, and HF is the set of Hash functions. For any k∈K, there is $h_{k}\in HF$, $h_{k}:O\to Z$.*

**Definition** **2.** *Hash function [23,24] $h\left(\cdot \right)\in HF$ is defined such that $\forall o,o^{\prime }\in O$, where $z=h\left(o\right)$ and $z^{\prime }=h\left(o^{\prime }\right)$, $o\neq o^{\prime }$ makes $z\neq z^{\prime }$.*

If o expressing the data need be transmitted via the communication channel, the information abstract z will be sent together with the data. After arriving at the receiving end, the information abstract of o (denoted as $\bar{z}$) is calculated. $z=\bar{z}$ means there was no change in o. However, there is a collision problem, which needs be verified as follows: $\exists o,\overset{⌢}{o}\in O$, where $z=h\left(o\right)$ and $\overset{⌢}{z}=h\left(\overset{⌢}{o}\right)$. $o\neq \overset{⌢}{o}$ and $z=\overset{⌢}{z}$ can be found.

**Theorem** **1.** *The probability of the collision problem is $1-\left(\frac{\left|Z\right|-1}{\left|Z\right|}\right)\left(\frac{\left|Z\right|-2}{\left|Z\right|}\right)\cdots \left(\frac{\left|Z\right|-\left|O\right|+1}{\left|Z\right|}\right)$, where $\left|\cdot \right|$ represents the amount of the elements [23,24].*

The common Hash function with secret keys means that the process of calculating $h\left(\cdot \right)$ needs to be controlled using secret keys, such as HMACSHA1 [25]. The Common Hash function only provides abstracts according to the algorithm. The Common Hash function will also be directly called the Hash function in this work. The Secure Hash Algorithm (SHA) is the most popular Hash function, and it is based on the Message-Digest Algorithm 4 (MD4) and Message-Digest Algorithm 5 (MD5) [26]. 

### 2.2. Encryption Technology of Symmetric Key

Encryption technology has two main categories, namely symmetric and unsymmetric keys. Symmetric keys were employed in this work, which means that encryption and decryption used the same key. The Data Encryption Standard (DES) is the most popular method because of its balance between accuracy and computational cost [27]. The general details of DES are as follows:

✧ Plain language is partitioned into several blocks of 64 bits in length.
✧ The length of an original key is also 64 bits, of which 8 bits are parity check bits. The original key is employed to construct a temp key in each calculation iteration.
✧ We set the current block to be CB. After the replacement of the current block, CB is partitioned into CB-I and CB-T subblocks of equal length.
✧ The operations of CB-I and CB-T in each iteration are different to balance the computation complexity and security of the encrypted data.

## 3. Methodology of Secure and Convenient Online Medical Consultations

COVID-19 imposed a new medical requirement, which decreased the frequency of face-to-face meetings during the epidemic. At the same time, in special situations, a patient may need the medical advice of doctors who are not located in the same region. Thus, it is necessary to build a complete scheme to solve these problems, and the quick development of computer technology makes this scheme possible. This section provides a solution. 

### 3.1. Encryption Dataflow

**Figure 1:**
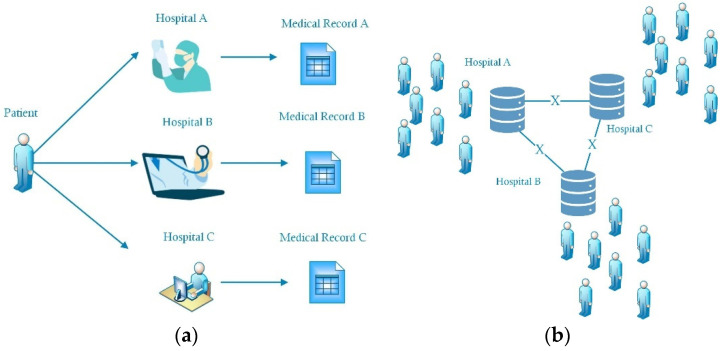
Schematic of medical record: (**a**) personal record; (**b**) hospital dataset.

**Figure 2:**
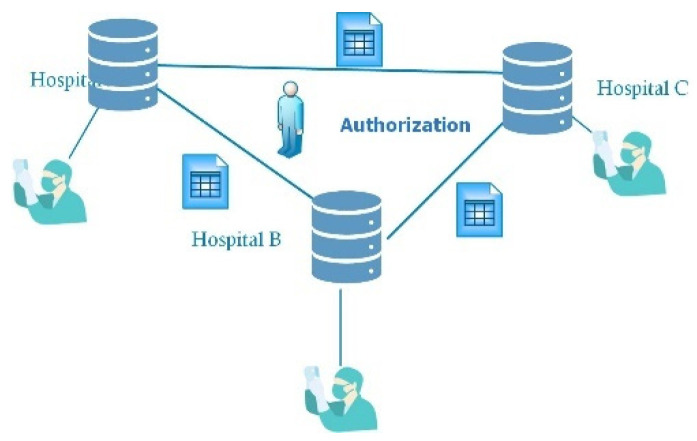
Legal sharing scheme.

When a patient need to be diagnosed and given treatment by doctors working at different hospitals, it is not convenient to employ hard copies of the patient’s medical history. Electronic medical records can easily be shared; however, the mechanism of their delivery and management must be built, as the privacy of patients must be strictly protected. Firstly, the difference between hard and electronic copies had to be analyzed. When the same patient attended three different hospitals, there were three different medical records, which could not be directly shared by the doctors. Sharing could only be accomplished if the patient brought the hard copy provided by each hospital to share their medical records. This method required a lot of time, which is very precious for patients, especially when the patient needs an integrated medical diagnosis from doctors working in different hospitals. The reason for this situation is that there are no safe and reliable sharing mechanisms for medical records. Every hospital has a dataset of medical records, which forms the basis of building a sharing mechanism. It is not possible to share all materials in the dataset between different hospitals, as shown in Figure 1a,b, because the privacy rights of the patients must be protected. The sharing process should satisfy the following rules, which are also described in Figure 2:

✧ Each delivery of a medical record needs the authorization of the patient;
✧ The delivery through public networks must be safe;
✧ Only the staff responsible for the patients have a right to see the shared documents.

In summary, the issues of personnel management and information security need to be solved. Personnel management involves obtaining authorization from the patients and defining the appropriate staff. Information security makes use of computer technology to secure the transmission of medical documents. Conventional authorization needs the signature of a certifier. With the help of computer technology, it was easy to solve this problem using the matched key. At the same time, encryption ensured that people without the matched key could not interpret the document. Thus, it was possible to create a reasonable mechanism to organize online medical conferences to help patients when receiving a face-to-face consultation was difficult. However, there was a very interesting finding. The patients who needed online medical meetings were not high-frequency users. If the keys used in the encryption system were based on patients, the identification of the user required additional costs in terms of money or time. In order to avoid additional costs, a third-party organization, namely the Online Medical Center (OMC), which has strong credibility, could undertake the allocation of keys or medical records belonging to the related patient. In the encryption system, the trusted organization was very generally able to deal with the task of verification. Moreover, in this special situation, the information holder had no right to directly provide the medical record, since this process made the communication process too complex, and the encryption channel connecting the information holder with the user could not be built. 

**Figure 3:**
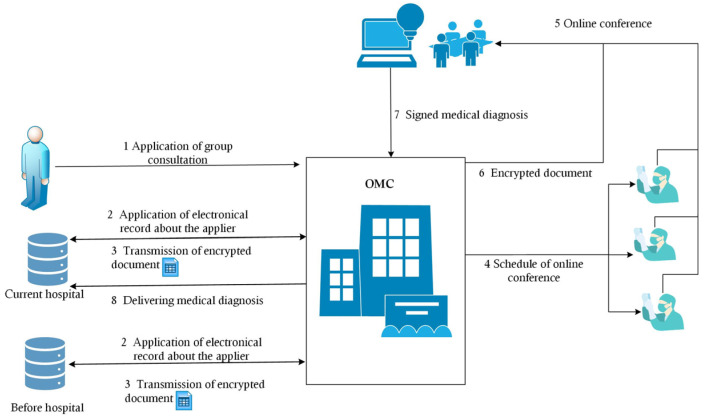
Dataflow of SMED.

In this work, a complete online mechanism was proposed to achieve the goal of supporting an online consultation with doctors in which a patient did not need to collect their medical records from different hospitals. This mechanism could reduce the costs related to communication, traffic, and time. Based on the above analysis, the main problems were the security and authorization of medical data. Through the help of encryption technology, a Special Medical Encryption Dataflow (SMED) was proposed to solve these problems. The flow diagram elaborating the steps of SMED is shown in Figure 3. 

**Methodology 1**—Special Medical Encryption Dataflow (SMED)

*Step 1: According to the suggestion of a doctor, the patient submits an application form to receive online medical group consultation, which includes a list of medical record holders and doctors taking part in the online conference.*

*Step 2: The OMC sends an application to collect the medical records of the patient to the hospitals mentioned on the list submitted by the patient.*

*Step 3: Hospitals deliver the encrypted data to the OMC.*

*Step 4: The OMC schedules the online conference, including the doctors and any required staff.*

*Step 5: The OMC provides the cryptographic materials to the list of participants.*

*Step 6: The doctors taking part in the conference decode the materials using their keys according to the defined methods. At the end of the conference, the medical diagnosis is given to the OMC.*

*Step 7: The OMC encrypts the diagnosis and sends it to the hospital at which the patient is currently being treated.*

### 3.2. Transmission Protocol

**Figure 4:**
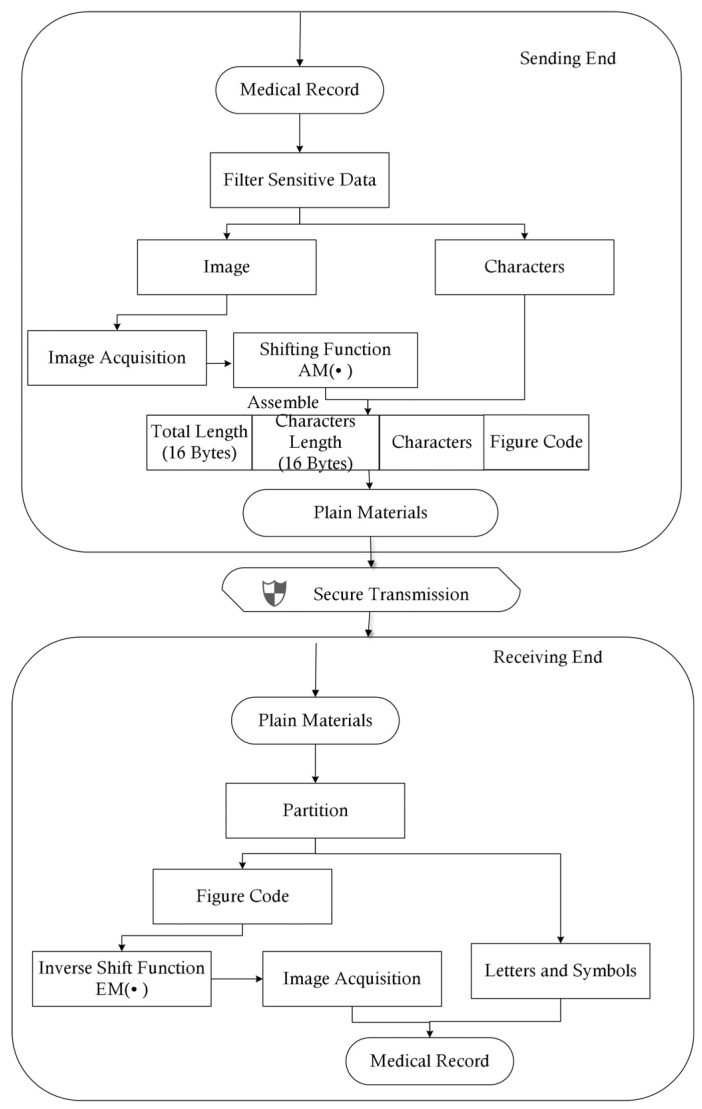
Flow Diagram of SMICTP.

The medical record contained personal information, such as personal IDs or addresses, which were not necessary for group consultations. Thus, before all of the data were transmitted via the encrypted channel built using the public networks, these sensitive data were filtered out. The formats of the medical records obtained from different hospitals or medical instruments may have been different; thus, the coding of computer technology was used to unify the transmission format. We defined the Secure Medical Image and Character Transmission Protocol (SMICTP) to complete this step. The construction of SMFCTP can be found in Methodology 2, the flow chart of which is shown in Figure 4. SMICTP was a little different from other medical transmission protocols. General medical transmission protocols often focus on one kind of medical data or do not distinguish catalogs of data [28,29], meaning that they miss useful information. However, the target of SMICTP was the design of reasonable language, for both characters and figures, used in Internet communications to ensure security and reduce costs. SMICTP, as a transmission protocol, coded the data as an information package at the sending end; when the packages arrived at the receiving end, the data were reassembled as complete data. Thus, the format of the sending end was initially given. To find the errors of transmission, SMICTP used a double-coding system in which medical images were independent of letters and symbols. This model allowed the verification of its own Hash function to provide a double check to determine data completeness. Besides this process, letters and symbols could directly use ASCII code; however, medical images needed to be interpreted before they could be transmitted by the network. Image Acquisition [30] was employed to divide images into three color channels. 

The overlapping of the codes between the figure and letter may have allowed additional decoding errors. Thus, the Shifting Function was defined.

**Definition** **3.** *The Shifting Function for figures was defined as follows:*

(1) $$AM\left(X\right)=\left\{\begin{array}{c} AM\left(X_{1}\right)=\Gamma_{k}\left(X_{1}+\omega ,\beta \right)=\left[x_{11}+\omega ,x_{12}+\omega ,\cdots ,x_{1k}+\omega \right],\\ \;\cdots ,\left[x_{1\left(nk+1\right)}+\omega ,x_{1\left(nk+2\right)}+\omega ,\cdots ,\beta \right]\\ AM\left(X_{2}\right)=\Gamma_{k}\left(X_{2}+\omega ,\beta \right)=\left[x_{21}+\omega ,x_{22}+\omega ,\cdots ,x_{2k}+\omega \right],\\ \;\cdots ,\left[x_{2\left(nk+1\right)}+\omega ,x_{2\left(nk+2\right)}+\omega ,\cdots ,\beta \right]\\ AM\left(X_{3}\right)=\Gamma_{k}\left(X_{3}+\omega ,\beta \right)=\left[x_{31}+\omega ,x_{32}+\omega ,\cdots ,x_{3k}+\omega \right],\\ \;\cdots ,\left[x_{3\left(nk+1\right)}+\omega ,x_{3\left(nk+2\right)}+\omega ,\cdots ,\beta \right] \end{array}\right.$$

*where* X *expresses a figure,* $AM\left(\cdot \right)$ *is its plain coder*, *and* $AM\left(X\right)=\left\{\begin{array}{c} AM(X_{1})\\ AM(X_{2})\\ AM(X_{3}) \end{array}\right.$ *expresses the color channels of the figure*. $X_{i}+\omega =\left\{x_{ij}+\omega ,x_{ij}\in X_{i}\right\}$. $\Gamma_{k}\left(\cdot ,\beta \right)$ *is the group function where one group has* k *elements, and if the amount of elements in the last group is less than* k*, the special value* β *is set as the complement.* ω *is a constant used to distinguish the code of figures from letters and symbols. Here, *$k=\frac{\tau }{\min\left(2,\left\lceil \frac{\mathrm{Max}\left(x_{ij}\right)+\omega }{255}\right\rceil \right)*8}$*, where* τ *is the number of bits in each group.*

At the receiving end, the code for the figures is re-translated via the Inverse Shift Function as follows:

(2) $$EM\left(X\right)=\left\{\begin{array}{c} \psi_{r}\left(\cup \left[-\beta ,X_{1}-\omega \right]\right)\\ \psi_{r}\left(\cup \left[-\beta ,X_{2}-\omega \right]\right)\\ \psi_{{}_{r}}\left(\cup \left[-\beta ,X_{3}-\omega \right]\right) \end{array}\right.$$

where $\cup \left[-\beta ,X_{1}-\omega \right]$ means that the complement β is initially deleted, and the code minus ω in each group is linked as one variable. $\psi_{r}\left(\cdot \right)$ splits the variable from a one-dimensional format into a two dimensional format with r rows. 

Besides the coding of figures and characters, 32 bytes were employed to show the total length and length of letters and symbols, respectively, which ensure the completeness of transmission. Figure 4 shows the complete flow chart of SMICTP. 

**Methodology 2**—Secure Medical Image and Character Transmission Protocol (SMICTP)

*Step 1: The actions at the sending end;*
  1.1 *Filter sensitive data from the electronic medical record.*
  1.2 *Obtain raw image code from Image Acquisition. Then, the Shifting Function is employed to provide the final image code.*
  1.3 *Calculate the length of figures codes and characters and the length of letters and symbols.*
  1.4 *Form the material package in the form of plain code. Set the length of all codes as the first two bytes and the length of letters and symbols as the third and fourth bytes. Then, all of the codes are at the end.*
*Step 2: Transmit the message via a secure channel;*
*Step 3: The actions at the receiving end.*
  3.1 *Read the first four bytes of the message to obtain the information to partition the image code and character.*
  3.2 *The Inverse Shift Function interprets the code for the figures to Image Acquisition, which redraws the medical figures based on the code.*
  3.3 *Obtain the letters and symbols.*
  3.4 *The electronic medical record is recovered.*

SMICTP provided the transmission flow of the medical records, where step 2 was the transmission through public networks. Step 2 was the most important part of SMICTP; moreover, it was very complex and needed a detailed description. Thus, Step 2 is introduced in the following subsection. 

### 3.3. Secure Channel

Methodology 1 describes the process of an online conference; Methodology 2 outlines a secure transmission framework for medical records. This section elaborates the details of the secure encryption technology, which supports these methodologies. Steps 3 and 8 of Methodology 1 needed an encrypted communication channel to connect the hospitals and OMC, which was called channel-HT. Steps 6 and 7 of Methodology 1 needed an encrypted communication channel to connect the doctors and OMC, which was called channel-DT. The content and theory of these two channels are completely different. 

**Figure 5:**
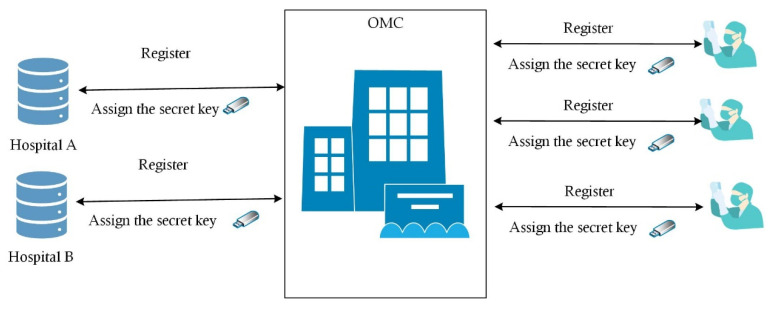
Allocation of keys.

On the sending or receiving end in Channel-HT was one user, i.e., one endpoint encrypted the data, and the other endpoint decoded the data. However, the number of doctors in channel-DT was more than one, as channel-DT had the responsibility to verify the authorization from the doctors to unpack the medical information. According to the application, more than one doctor was enough to secure authorization. The difficulty was that the number of users was not certain. At the same time, the endpoints of the channels were the OMC, hospitals, and doctors, all of which frequently took part in online conferences. Hospitals and doctors only needed to register in the OMC to obtain their own key. Then, the key did not need to be allocated to carry out every group consultation, as shown in Figure 5. It must be noted that the keys allocated to doctors who attended the same online conference had to satisfy the rule, which will be introduced in the following theory.

**Figure 6:**
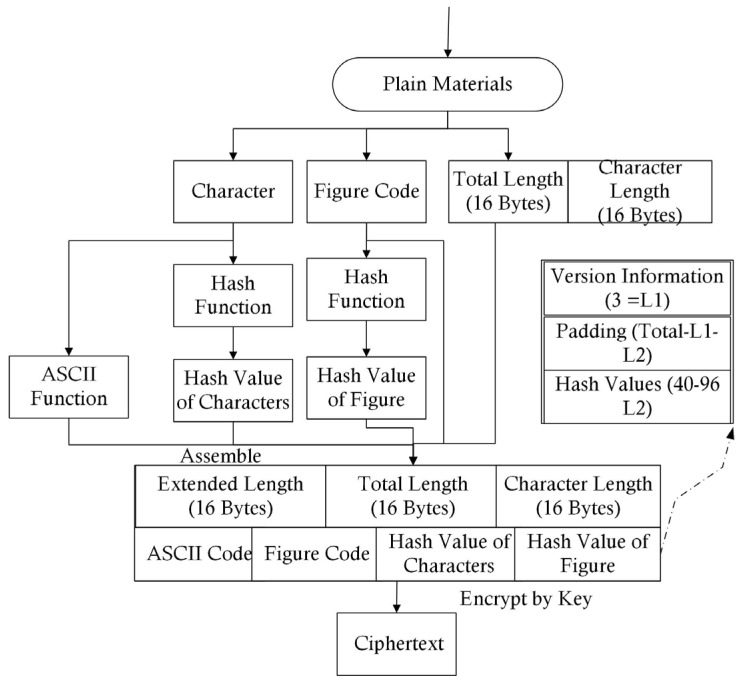
Package at sending end.

Channel-HT employs the theory of symmetrical encryption to perform the secure communication between the hospitals and OMC. Two targets had to be be met: (1) the transmitted data had to be in the format of ciphertext, and (2) the sender of the data had to be verified. Target (1) was easy to understand as the protection of the medical records. Target (2) prevented the transmitted data from being intercepted and forged. In order to realize these targets, the character and figure code were specifically calculated to obtain a Hash value, which was added to the materials, because the information abstract given by Hash function ensured data completeness. Completeness means that the data will not be changed during the transmission process. The Hash function has the ability to verify the completeness because of its irreversible characteristic. After the Hash values were added, the extended length was updated to form the raw material of the ciphertext. The private key was employed to encrypt the materials. This process is shown in Figure 6. It should be noted that the data were transmitted as ciphertext to protect the private information of patients. This work used SHA as a standard to calculate the Hash value. Each version of SHA set its own length of information abstract. And in order to simplify the process of encryption, the Hash values were decoded as characters. Thus, 128 bytes were the union length, among which the first 3 bytes were the version information. The Hash values were located at last, and the others were filled with special digits. 

**Figure 7:**
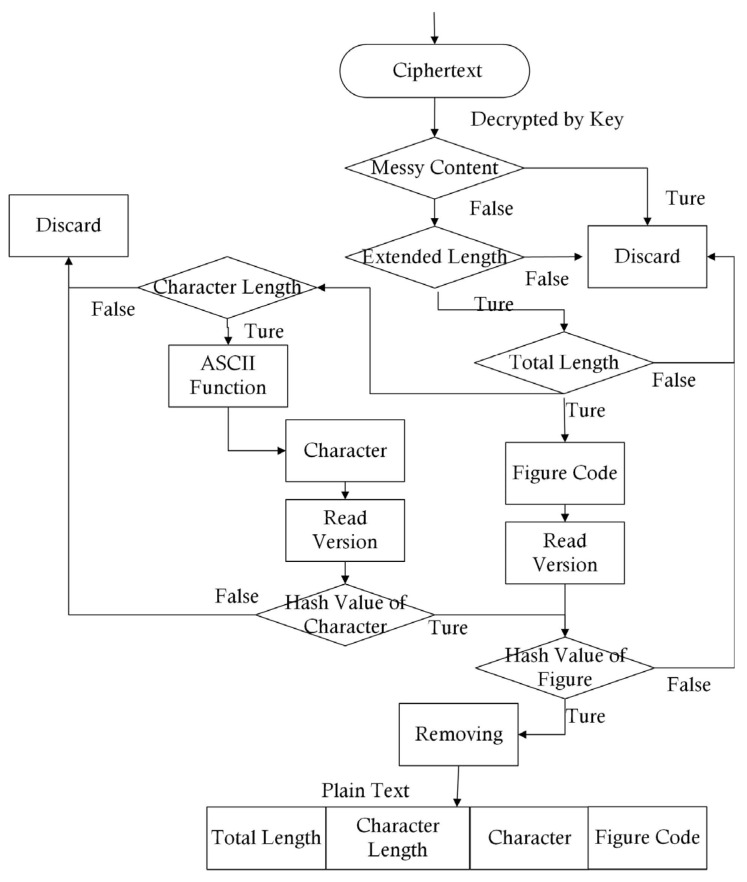
Package at receiving end.

When the transmitted information arrived, the receiver used the private key to decipher the data. Since the keys were allocated to the sender and receiver in a symmetrical format, this becomes the true digital signature. Thus, the pair of keys verified the source of the data. Then, the correctness of the data had to be verified. Based on the format of the data, a double guarantee could be found. Firstly, the data had three different definitions of length, which were compared to their prior values. This simple and fast step measured whether the data were changed during the communication process. These changes could include errors from the public network or falsification. Secondly, if the data had been changed, the Hash value calculated at the arriving end could not be equal to the fixed value in the data package (Figure 7). After identifying the clear targets of package, Methodology 3 (Complete and Safe Medical Package (CSMP)), the input of which was derived from Methodology 2, was introduced as follows. 

**Methodology 3**—Complete and Safe Medical Package (CSMP) 

*Sending end*

*Step 1: Form the Hash value. The character and figure code are inputted into the Hash function to obtain their information abstracts. Then, the version information of the employed Hash function is written in the first three locations. The others are filled with a special value.*

*Step 2: The extended length is defined as the addition of the total length and two Hash values. Then, we assemble the extended length and Hash values as a data package in plain language.*

*Step 3: The data package is encrypted using symmetrical keys.*

*Receiving End*

*Step 1: When a new package arrives, the data are encrypted using the paired key. If the data are in the format of messy content, the data are discarded, and the sender is asked to retransmit the data.*

*Step 2: The extended length, total length, and character length are calculated to compare them to their corresponding values in the package. Any unequal comparisons lead to the discarding of the package.*

*Step 3: The information abstracts of the character and figure code are calculated based on the version information of the Hash function found in the first three locations. Then, these values are compared to the values found in the package. Any unequal comparison leads to the discarding of the package.*

*Step 4: The extended length and two Hash values are removed from the package. The remaining part is the plain text of the received package.*

Channel-DT defined its own communication principle, which was different from that of Channel-HT.

Each communication through Channel-HT has only two participants; however, generally, each communication through Channel-DT has more than two participants. Moreover, it provided an option to approve the unpacking of the medical record. More than one doctor taking part in online conference could give permission; the other option needed the permission of all doctors participating in the conference. Thus, the novel principle of Channel-DT was needed. Before building Channel-DT, some basic theory was introduced [24,31].

**Definition** **4.** *Define a positive integer set $S=\left\{s_{1},s_{2},\cdots ,s_{t}\right\}$ [24,31]. $\forall s_{i},s_{j}\in S$, if i≠j, and $s_{i},s_{j}$ are relatively prime, being expressed as $\gcd\left(s_{i},s_{j}\right)=1$. For a positive integer set $P=\left\{p_{1},p_{2},\cdots ,p_{t}\right\}$, the congruence equation of integer o is defined as follows:*

(3) $$\begin{array}{l} o\equiv p_{1}\left(\mathrm{mod}s_{1}\right)\\ o\equiv p_{2}\left(\mathrm{mod}s_{2}\right)\\ \;\vdots \\ o\equiv p_{t}\left(\mathrm{mod}s_{t}\right) \end{array}$$

*$o\equiv p_{i}\left(\mathrm{mod}s_{i}\right)$ *means that* $p_{i}$ *is the remainder when* o *is divided by* $s_{i}$.*

**Definition** **5.** *If set $\tilde{S}=\prod_{s_{i}\in S}s_{i}$, for each $s_{i}$, define $y_{i}={\tilde{s}}_{i}^{-1}\mathrm{mod}s_{i}$, where ${\tilde{s}}_{i}=\frac{\tilde{S}}{s_{i}}$, and mod is the remainder function. $y_{i}={\tilde{s}}_{i}^{-1}\mathrm{mod}s_{i}$ means that $y_{i}$ is the remainder when ${\tilde{s}}_{i}^{-1}$ is divided by $s_{i}$. Thus, $\left({\tilde{s}}_{i}*y_{i}\right)\mathrm{mod}s_{i}=1$ [24,31].*

**Theorem** **2.** *$\forall s_{i},s_{j}\in S$, if i≠j, and $s_{i},s_{j}$ are relatively prime, and a positive integer set $P=\left\{p_{1},p_{2},\cdots ,p_{t}\right\}$, the congruence equation of integer o, has a unique solution, which is $o=\sum_{i=1}^{t}p_{i}{\tilde{s}}_{i}y_{i}\mathrm{mod}\tilde{S}$. This situation is known as the Chinese Remainder Theorem [31].*

Due to the different options used to unpack the medical record, Theorem 2 could not be used directly. Based on the theory, an extended theorem was proposed. The hardest part of this problem is that any two doctors had ability to give permission, which could not be foreseen before the online conference, for the committee to agree that more than one doctor was enough to unpack the medical record. Also, there was another choice, that permission could only be given if all doctors gave authorization. The extended theorem had to be included in these cases.

**Definition** **6.** *If set $S^{\prime }=k\times \prod_{s_{i}\in S}s_{i}+\left(1-k\right)\times \prod_{s_{i}\in S}\left(s_{i}\times \max\left(l_{i},\frac{1}{s_{i}}\right)\right)$, for each $s_{i}$, define $y_{i}=s_{i}^{\prime -1}\mathrm{mod}s_{i}$, where $s_{i}^{\prime }=\frac{S^{\prime }}{s_{i}}$ and $k,l_{i}\in \left\{0,1\right\},i=1,2,\cdots ,t$. $l_{i}$ is known as person the parameter.*

**Theorem** **3.** *$\forall s_{i},s_{j}\in S$, if i≠j, and $s_{i},s_{j}$ are relatively prime, and there is a positive integer set $P=\left\{p_{1},p_{2},\cdots ,p_{t}\right\}$, the congruence equation of integer o in the extended situation has the unique solution $\hat{o}=\sum_{i=1}^{t}\left(1-k\right)\left(l_{i}p_{i}s_{i}^{\prime }y_{i}\mathrm{mod}S^{\prime }\right)+k\sum_{i=1}^{t}p_{i}s_{i}^{\prime }y_{i}\mathrm{mod}S^{\prime }$. This is called as Extended Chinese Remainder Theorem.*

**Proof** **of** **Theorem 3.** Define $F=\left\{f_{j}|,f_{j}=s_{i,}\;if\;l_{i}\neq 0,1\leq i\leq t\right\}$ and let $g=\left|F\right|$, where $\left|\cdot \right|$ denotes the cardinality of the set. Thus, 1≤j≤g.

(1) k=0

Based on the set F, the congruence equation of integer o is $\begin{array}{l} o\equiv h_{1}\left(\mathrm{mod}f_{1}\right)\\ o\equiv h_{2}\left(\mathrm{mod}f_{2}\right)\\ \;\vdots \\ o\equiv h_{g}\left(\mathrm{mod}f_{g}\right) \end{array}$. It is easy to obtain $H=\left\{h_{i}|h_{i}=p_{j}\;if\;f_{i}=s_{j}\right\}$ because of the same digits in the equations. According to Theorem 2, the unique solution is $o=\sum_{i=1}^{g}h_{g}{\tilde{f}}_{i}z_{i}\mathrm{mod}\tilde{F}$, where $\tilde{F}=\prod_{f_{i}\in F}f_{i}$, ${\tilde{f}}_{i}=\frac{\tilde{F}}{f_{i}}$, and $z_{i}={\tilde{f}}_{i}^{-1}\mathrm{mod}f_{i}$.

$S^{\prime }=k\times \prod_{s_{i}\in S}s_{i}+\left(1-k\right)\times \prod_{s_{i}\in S}\left(s_{i}\times \max\left(l_{i},\frac{1}{s_{i}}\right)\right)=\prod_{s_{i}\in S}\left(s_{i}\times \max\left(l_{i},\frac{1}{s_{i}}\right)\right)$ in Definition 6 because of k=0. Then, $s_{i}\times \max\left(l_{i},\frac{1}{s_{i}}\right)=s_{i}\times \frac{1}{s_{i}}=1$ if $l_{i}=0$. Thus, it can be concluded that $S^{\prime }=\prod_{s_{i}\in S\wedge l_{i}=1}s_{i}$. $F=\left\{f_{j}|,f_{j}=s_{i,}\;if\;l_{i}\neq 0,1\leq i\leq t\right\}\Rightarrow \tilde{F}=S^{\prime }$.

The equation in Theorem 3 shows that $\hat{o}=\sum_{i=1}^{t}\left(1-k\right)\left(l_{i}p_{i}s_{i}^{\prime }y_{i}\mathrm{mod}S^{\prime }\right)+k\sum_{i=1}^{t}p_{i}s_{i}^{\prime }y_{i}\mathrm{mod}S^{\prime }$ because of k=0 and $\hat{o}=\sum_{i=1}^{t}l_{i}p_{i}s_{i}^{\prime }y_{i}\mathrm{mod}S^{\prime }$. Moreover, $l_{i}=0\Rightarrow l_{i}p_{i}s_{i}^{\prime }y_{i}\mathrm{mod}S^{\prime }=0.$ When $\forall l_{j}=1$, $\exists f_{j}=s_{i}\;and\;\tilde{F}=S^{\prime }\Rightarrow {\tilde{f}}_{j}=s_{i}{}^{\prime },z_{j}=y_{i}$ and $h_{i}=p_{j}$, $\hat{o}=o$.

(2) k=1

$S^{\prime }=\prod_{s_{i}\in S}s_{i}$ and $\overset{⌢}{o}=\sum_{i=1}^{t}p_{i}s_{i}^{\prime }y_{i}\mathrm{mod}S^{\prime }$ which can be directly verified by Theorem 2. □

When all doctors needed to give authorization, k=1. According to the proof of situation (2), Theorem 3 was equal to Theorem 2. If the conference committee only needed the approval of more than one doctor to unpack the documents, k=0. $\hat{o}$ was the plain language, and $P=\left\{p_{1},p_{2},\cdots ,p_{t}\right\}$ was the set of ciphertext allocated to every doctor take part in the online conference. $S=\left\{s_{1},s_{2},\cdots ,s_{t}\right\}$ was the set of authentication keys belonging to every doctor. In the initial setting, $L=\left\{l_{i}=0,1\leq i\leq t\right\}$. For those for whom keys were available, the related person parameter was set at 1($l_{i}=1$). 

The authentication key was also transmitted to approve the unpacking of the medical record through the public network, which needed to be protected. Every doctor had a paired key to encrypt the documents. It was convenient to directly use the paired key to encrypt the authentication key.

**Definition** **7.** *Key package includes two keys expressed as $\left\{\mathrm{Key}\text{-}P,\mathrm{Key}\text{-}C\right\}$. Key-P is one of the paired keys. Key-C is an authentication key. Con-key means that Key-C is encrypted using Key-P.*

**Figure 8:**
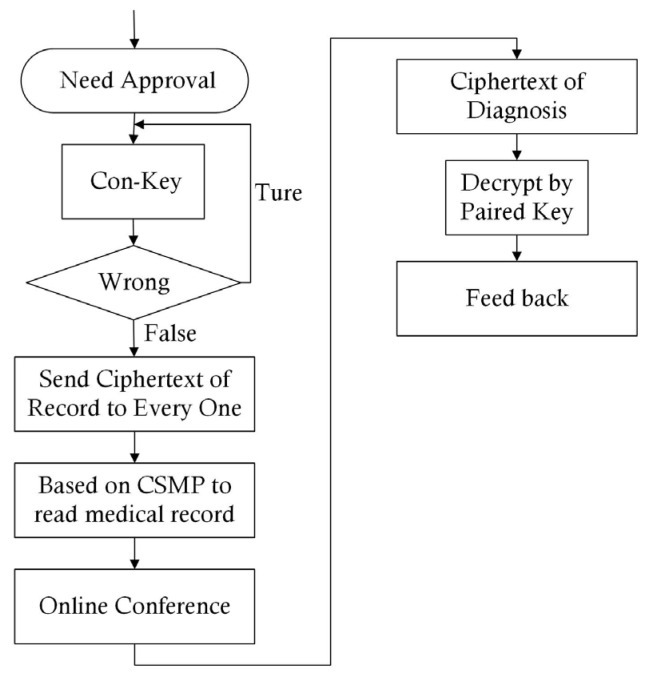
Chart of SPMOC.

Thus, the doctors provided the Con-key to approve the unpacking of the medical record. When the OMC received the Con-key, the key was first decrypted by the paired key to obtain the real Key-C. Then, Key-C was employed according to Theorem 3. The details of Methodology 4 (Secure Protocol of Medical Online Conference (SPMOC)) are described in Figure 8.

**Methodology 4**—Secure Protocol of Medical Online Conference (SPMOC) 

*Step 1: The conference committee puts the ciphertext of the authorization digits in the online server.*

*Step 2:* *The doctors use the Con-key to decrypt the ciphertext to provide the authorization according to Theorem 3. If the digits are correct, the process moves to step 3; otherwise, the process moves back to Step 1.*

*Step 3: After authorization, the encrypted medical document is sent to the participants of the conference, the format of which is designed as shown Methodology 3. When the materials arrive, the Key-P* *of each person is employed to decrypt the documents.*

*Step 4: Hold the online conference.*

*Step 5: After the OMC handles the ciphertext of the diagnosis that has been encrypted using the Key-P* *paired with every doctor, send it back to theresponsible hospital of the patient.*

*Step 6: The hospital decrypts the final result and then gives feedback.*

It was found that Step 2 of Methodology 4 used the Con-key to give authorization based on Theorem 3. Thus, it was necessary to explain this process in detail. The core purpose of Step 2 was to give the authorization required to unpack the medical record. Theorem 3 solved this problem as follows: When $P=\left\{p_{1},p_{2},\cdots ,p_{t}\right\}$ is deployed in the conference, the doctor can send the Key-C, which corresponds to $s_{i}\in S$, to the server. Then, according to Theorem 3, the correct digits can be obtained. However, Key-C cannot be sent through the internet; thus, Key-C is encrypted by Key-P, which is defined as Con-key. When the Con-key arrives at the conference server, it is decoded by the paired key, which is also used in following communications. 

The above content constructed a virtual private chain to finish a secure medical online conference. This private chain contained the participants as nodes and secure channels as links. The plain codes of the package, including original information, Hash values, and length digits, had the ability to recognize any modification during transmission process due to network errors or attacks, which were introduced in Methodologies 3 and 4. This process ensured the completeness of medical data. And patients, doctors, OMC, and medical copy holders, as participants in online medical conferences, had their own authority controlled by the allocation of encryption keys. After the package was encrypted by the secret keys, the ciphertext was transmitted through a public network. Any illegal reading and modification was totally limited by encryption and correctness checks. Thus, a temporary private chain able to organize an online medical conference was generated through public networks.

## 4. Case Elaboration 

In this section, a classical case is given to show a complete process of holding an online conference according to the proposed framework and encryption methods. This case sets a scene of a patient who needs medical advice from doctors who live in different regions. In order to obtain rapid medical care, the patient sends an application to the OMC to hold an online conference. 

After submitting the application to hold an online conference, the OMC collects the related medical records listed on the application form according to SMED (Methodology 1), which gives the framework of the conference. It is reasonable that hospitals are the high-frequency users of the OMC; thus, the symmetrical key has been allocated to the hospitals. The medical record is packaged as the format defined by CSMP (Methodology 3), which can be transmitted through the public network in a secure way. After the symmetrical key is employed to encrypt the records, the encrypted package is transmitted to the OMC. The packages received by the OMC verify the parameters in terms of length in a quick manner. If the parameters are not equal to the values listed in the packages, the packages will be discarded. Moreover, information abstracts given by the Hash function, which are shown in CSMP, verify the completeness of the data during the communication. 

The medical materials are kept in the server of the OMC. When the conference starts, the doctors need to send their Con-keys to the server to provide the authorization required to unpackage the electronic records. The Con-key includes the Key-C encrypted using Key-P introduced in Definition 7. The Key-P is allocated to the doctor in a paired form, which is used to verify the identity and encryption of the data. When the server receives the Con-key, the process of authorization is verified according to the Extended Chinese Remainder Theorem. A successful authorization enables the data encrypted using Key-P to be sent to every doctor. After enough discussion, the staff of the OMC summarize and send the final results in the form of ciphertext, which is also calculated using Key-P. These results are given via SPMOC (Methodology 4). 

The core problems will be explained in the following subsection, which includes the medical record encryption, information abstract calculation using Hash function, and authorization based on the Extended Chinese Remainder Theorem.

### 4.1. Example—Authorization Based on ECRT

This subsection displays the calculation process using the Extended Chinese Remainder Theorem (Theorem 3 ECRT) to support the authorization under the condition of uncertain certifiers. As mentioned in the introduction, if all doctors taking part in the conference needed to provide their keys to unpack the medical record, this process was the same for Theorem 2. When some of the doctors were able to give authorization, ECRT was necessary to give the theory foundation of authorization. Moreover, the providers were uncertain before they sent their keys. The Con-key was employed in the process, which meant that Key-C was encrypted using Key-P. The encryption of Key-P will be shown in the following subsection. The repetition of paired keys is omitted to focus on the employment of Key-C.

The initial authorization is as follows: (1) Assume the plain language is o=116. (2) The allocated Key-C to each doctor is $Key\text{-}C_{1}=55$, $Key\text{-}C_{2}=23$, and $Key\text{-}C_{3}=41$. (3) The ciphertext allocated to three doctors are $p_{1}=116\mathrm{mod}55=6$, $p_{2}=116\mathrm{mod}23=1$, and $p_{3}=116\mathrm{mod}41=34$. The meaning of mod can be found in Definition 5.

(1) Doctors 1 and 2 provide the Con-keys; thus, k=0, $l_{1}=l_{2}=1$, and $l_{3}=0$. $Key\text{-}C_{1}=55,Key\text{-}C_{2}=23,Key\text{-}C_{3}=*\Rightarrow s_{1}=55,s_{2}=23,s_{3}=*$. According to Definition 6, $S^{\prime }$ is calculated using the method shown below.
  $$\begin{array}{ll} S^{\prime } & =k\times \prod_{s_{i}\in S}s_{i}+\left(1-k\right)\times \prod_{s_{i}\in S}\left(s_{i}\times \max\left(l_{i},\frac{1}{s_{i}}\right)\right)\\ & =0\times \left(55\times 23\times *\right)+\left(1-0\right)\times 55\times \max\left(1,\frac{1}{55}\right)\times 23\times \max\left(1,\frac{1}{23}\right)\times *\times \max\left(0,\frac{1}{*}\right)\\ & =1265 \end{array}$$

Then, $s_{1}^{\prime }=\frac{S^{\prime }}{s_{1}}=23$, $s_{2}^{\prime }=\frac{S^{\prime }}{s_{2}}=55$, and $s_{3}^{\prime }=\frac{S^{\prime }}{s_{3}}=\frac{S^{\prime }}{*}$; $y_{1}=s_{1}^{\prime -1}\mathrm{mod}s_{1}=23^{-1}\mathrm{mod}55=12$; and $y_{2}=s_{2}^{\prime -1}\mathrm{mod}s_{2}=55^{-1}\mathrm{mod}23=18$. The plain language, based on Theorem 3, can be defined as follows:

$$\begin{array}{ll} \hat{o} & =\sum_{i=1}^{t}\left(1-k\right)l_{i}p_{i}s_{i}^{\prime }y_{i}\mathrm{mod}S^{\prime }+k\sum_{i=1}^{t}p_{i}s_{i}^{\prime }y_{i}\mathrm{mod}S^{\prime }\\ & =\sum_{i=1}^{t}\left(1-0\right)l_{i}p_{i}s_{i}^{\prime }y_{i}\mathrm{mod}S^{\prime }+0\times \sum_{i=1}^{t}p_{i}s_{i}^{\prime }y_{i}\mathrm{mod}S^{\prime }\\ & =\left(\left(1-0\right)\left(1\times 6\times 23\times 12\right)+\left(1-0\right)\left(1\times 1\times 55\times 18\right)+\left(1-0\right)\left(0\times p_{3}s_{3}^{\prime }y_{3}\right)\right)\mathrm{mod}1265\\ & =116 \end{array}$$

(2) Doctors 1 and 3 provide the Con-keys; thus, k=0, $l_{1}=l_{3}=1$, and $l_{2}=0$. $Key\text{-}C_{1}=55,Key\text{-}C_{2}=*,Key\text{-}C_{3}=41\Rightarrow s_{1}=55,s_{2}=*,s_{3}=41$. According to Definition 6,
  $$\begin{array}{ll} S^{\prime } & =k\times \prod_{s_{i}\in S}s_{i}+\left(1-k\right)\times \prod_{s_{i}\in S}\left(s_{i}\times \max\left(l_{i},\frac{1}{s_{i}}\right)\right)\\ & =0\times \left(55\times *\times 41\right)+\left(1-0\right)\times 55\times \max\left(1,\frac{1}{55}\right)\times *\times \max\left(0,\frac{1}{*}\right)\times 41\times \max\left(1,\frac{1}{41}\right)\\ & =2255 \end{array}$$

Then, $s_{1}^{\prime }=\frac{S^{\prime }}{s_{1}}=41$, $s_{2}^{\prime }=\frac{S^{\prime }}{s_{2}}=\frac{S^{\prime }}{*}$, and $s_{3}^{\prime }=\frac{S^{\prime }}{s_{3}}=55$. $y_{1}=s_{1}^{\prime -1}\mathrm{mod}s_{1}=41^{-1}\mathrm{mod}55=51$, and $y_{3}=s_{3}^{\prime -1}\mathrm{mod}s_{3}=55^{-1}\mathrm{mod}41=3$. To work out plain language based on Theorem-3, we can use the following formula:

$$\begin{array}{ll} \hat{o} & =\sum_{i=1}^{t}\left(1-k\right)\left(l_{i}p_{i}s_{i}^{\prime }y_{i}\right)\mathrm{mod}S^{\prime }+k\sum_{i=1}^{t}p_{i}s_{i}^{\prime }y_{i}\mathrm{mod}S^{\prime }\\ & =\sum_{i=1}^{t}\left(1-0\right)\left(l_{i}p_{i}s_{i}^{\prime }y_{i}\right)\mathrm{mod}S^{\prime }+0\times \sum_{i=1}^{t}p_{i}s_{i}^{\prime }y_{i}\mathrm{mod}S^{\prime }\\ & =\left(\left(1-0\right)\left(1\times 6\times 41\times 51\right)+\left(1-0\right)\left(0\times 1\times p_{2}s_{2}^{\prime }y_{2}\right)+\left(1-0\right)\left(1\times 34\times 55\times 3\right)\right)\mathrm{mod}2255\\ & =116\; \end{array}$$

(3) Doctors 2 and 3 provide the Con-keys; thus, k=0, $l_{2}=l_{3}=1$ and $l_{1}=0$. $Key\text{-}C_{1}=*,Key\text{-}C_{2}=23,Key\text{-}C_{3}=41\Rightarrow s_{1}=*,s_{2}=23,s_{3}=41$. According to Definition 6,
  $$\begin{array}{ll} S^{\prime } & =k\times \prod_{s_{i}\in S}s_{i}+\left(1-k\right)\times \prod_{s_{i}\in S}\left(s_{i}\times \max\left(l_{i},\frac{1}{s_{i}}\right)\right)\\ & =0\times \left(*\times 23\times 41\right)+\left(1-0\right)\times *\times \max\left(0,\frac{1}{*}\right)\times 23\times \max\left(1,\frac{1}{23}\right)\times 41\times \max\left(1,\frac{1}{41}\right)\\ & =943 \end{array}$$

Then, $s_{1}^{\prime }=\frac{S^{\prime }}{s_{1}}=\frac{S^{\prime }}{*}$, $s_{2}^{\prime }=\frac{S^{\prime }}{s_{2}}=41$, and $s_{3}^{\prime }=\frac{S^{\prime }}{s_{3}}=23$. $y_{2}=s_{2}^{\prime -1}\mathrm{mod}s_{2}=41^{-1}\mathrm{mod}23=9$, and $y_{3}=s_{3}^{\prime -1}\mathrm{mod}s_{3}=23^{-1}\mathrm{mod}41=25$. To work out plain language based on Theorem 3, we can use the following formula:

$$\begin{array}{ll} \hat{o} & =\sum_{i=1}^{t}\left(1-k\right)\left(l_{i}p_{i}s_{i}^{\prime }y_{i}\right)\mathrm{mod}S^{\prime }+k\sum_{i=1}^{t}p_{i}s_{i}^{\prime }y_{i}\mathrm{mod}S^{\prime }\\ & =\sum_{i=1}^{t}\left(1-0\right)\left(l_{i}p_{i}s_{i}^{\prime }y_{i}\right)\mathrm{mod}S^{\prime }+0\times \sum_{i=1}^{t}p_{i}s_{i}^{\prime }y_{i}\mathrm{mod}S^{\prime }\\ & =\left(\left(1-0\right)\left(0\times p_{1}s_{1}^{\prime }y_{1}\right)+\left(1-0\right)\left(1\times 1\times 41\times 9\right)+\left(1-0\right)\left(1\times 34\times 23\times 25\right)\right)\mathrm{mod}943\\ & =116\; \end{array}$$

(4) All doctors provide the Con-keys; thus, k=1 and $l_{1}=l_{2}=l_{3}=1$. $C_{2}=23,Key\text{-}C_{3}=41\Rightarrow s_{1}=55,s_{2}=23,s_{3}=41$. According to Definition 6,
  $$\begin{array}{ll} S^{\prime } & =k\times \prod_{s_{i}\in S}s_{i}+\left(1-k\right)\times \prod_{s_{i}\in S}\left(s_{i}\times \max\left(l_{i},\frac{1}{s_{i}}\right)\right)\\ & =1\times \left(55\times 23\times 41\right)+\left(1-1\right)\times \prod_{s_{i}\in S}\left(s_{i}\times \max\left(l_{i},\frac{1}{s_{i}}\right)\right)=51865 \end{array}$$

Then, $s_{1}^{\prime }=\frac{S^{\prime }}{s_{1}}=\frac{S^{\prime }}{55}=943$, $s_{2}^{\prime }=\frac{S^{\prime }}{s_{2}}=\frac{S^{\prime }}{23}=2255$, and $s_{3}^{\prime }=\frac{S^{\prime }}{s_{3}}=\frac{S^{\prime }}{41}=1265$; $y_{1}=s_{1}^{\prime -1}\mathrm{mod}s_{1}=943^{-1}\mathrm{mod}55=7$; $y_{2}=s_{2}^{\prime -1}\mathrm{mod}s_{2}=2255^{-1}\mathrm{mod}23=1$; and $y_{3}=s_{3}^{\prime -1}\mathrm{mod}s_{3}=1265^{-1}\mathrm{mod}41=34$. To work out plain language based on Theorem 3, we can use the following formula:

$$\begin{array}{ll} \hat{o} & =\sum_{i=1}^{t}\left(1-k\right)\left(l_{i}p_{i}s_{i}^{\prime }y_{i}\right)\mathrm{mod}S^{\prime }+k\sum_{i=1}^{t}p_{i}s_{i}^{\prime }y_{i}\mathrm{mod}S^{\prime }\\ & =\sum_{i=1}^{t}\left(1-1\right)\left(l_{i}p_{i}s_{i}^{\prime }y_{i}\mathrm{mod}S^{\prime }\right)+1\times \sum_{i=1}^{t}p_{i}s_{i}^{\prime }y_{i}\mathrm{mod}S^{\prime }\\ & =\left(6\times 943\times 7+1\times 2255\times 1+34\times 1265\times 34\right)\mathrm{mod}51865\\ & =116\; \end{array}$$

### 4.2. Medical Record Encryption

**Figure 9:**
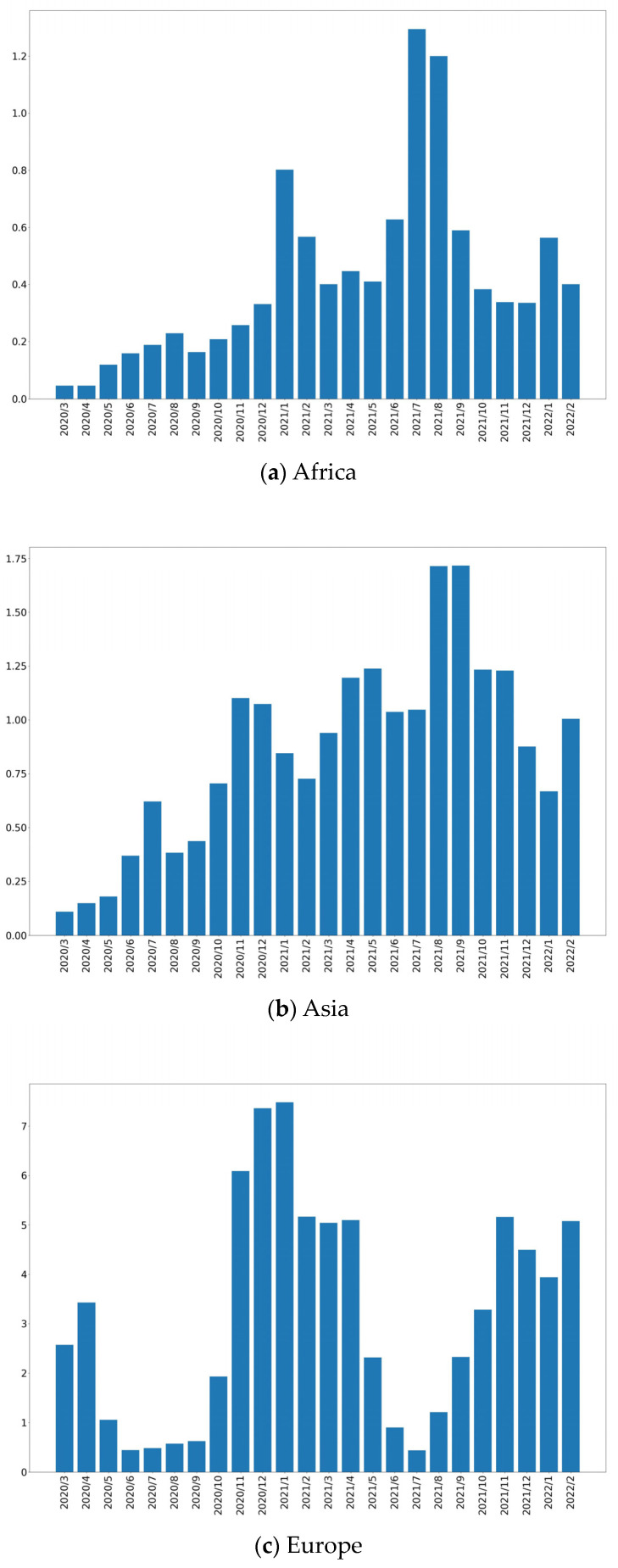

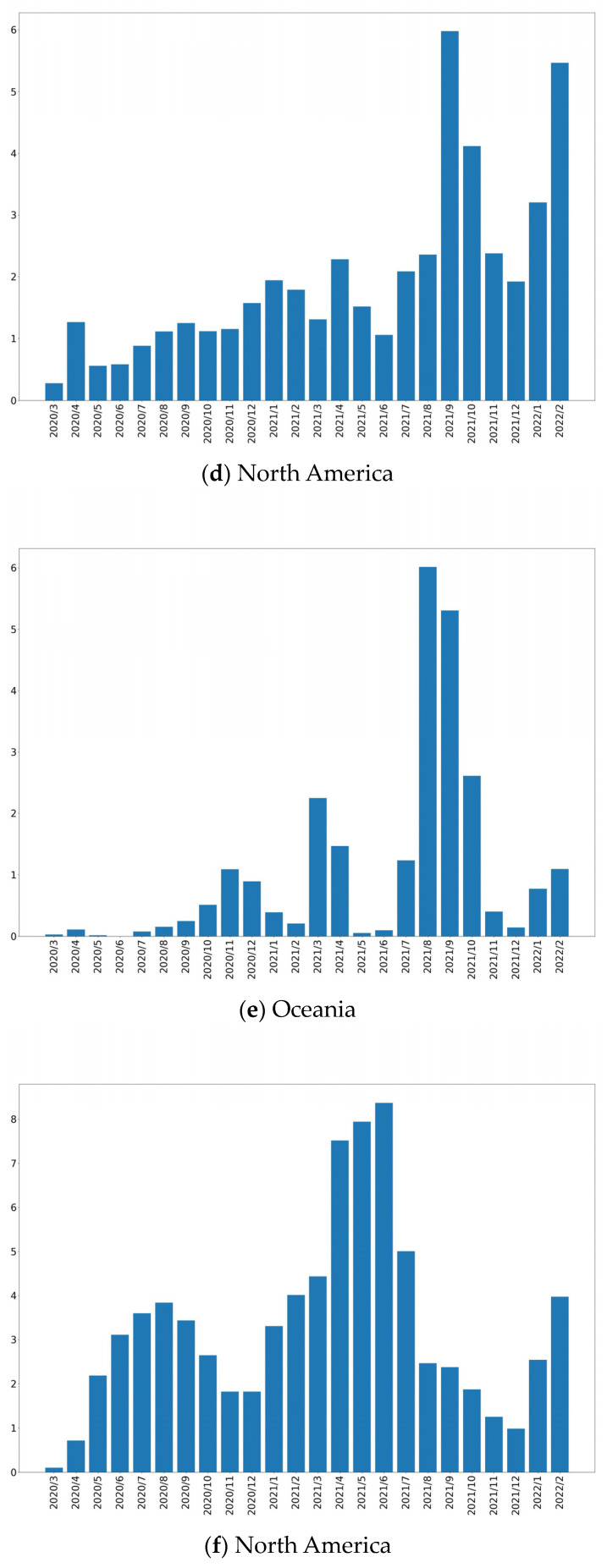
Mean values of new death cases per million.

**Figure 10:**
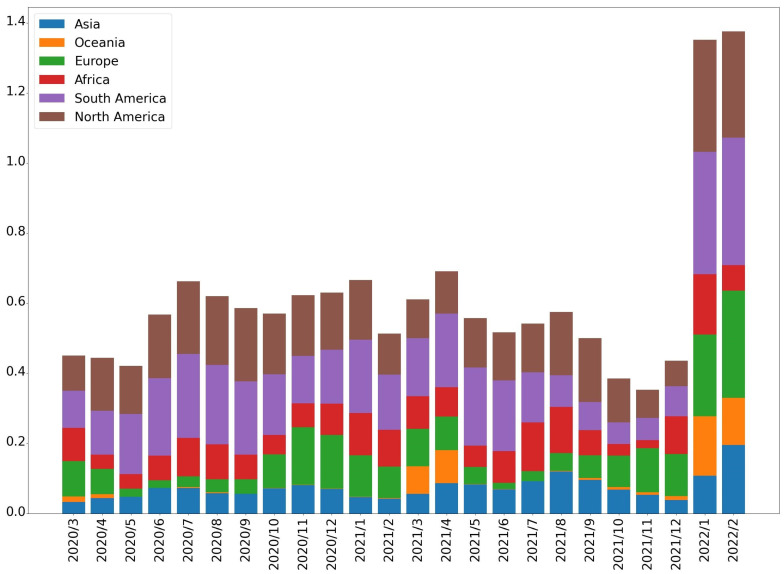
Result of testing.

**Figure 11:**
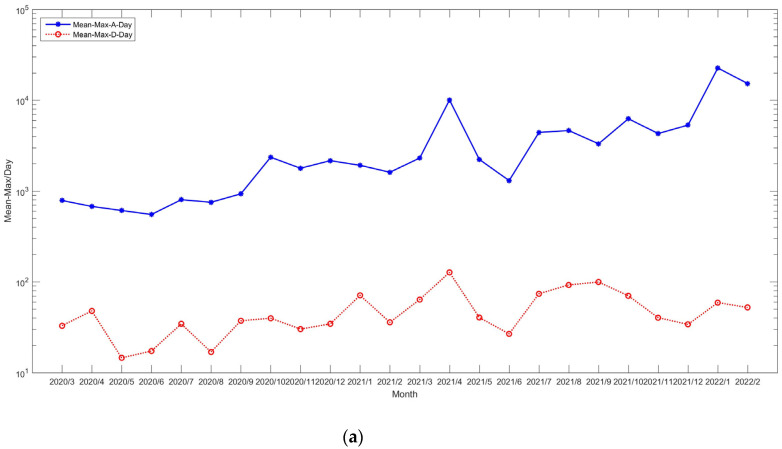

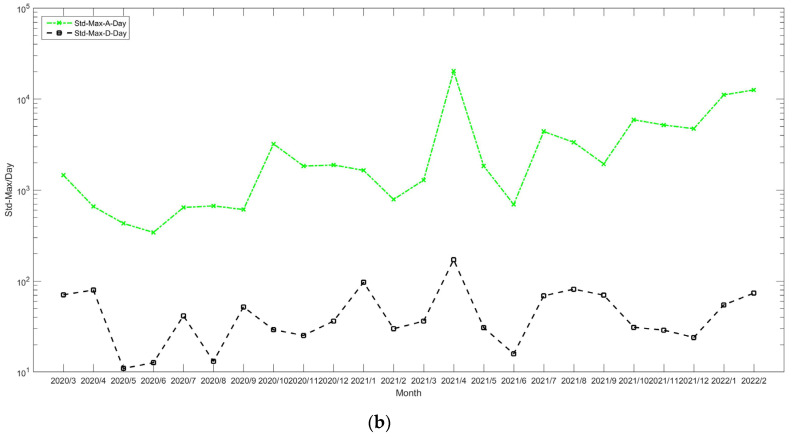
Statistical values: (**a**) mean function; (**b**) standard deviation.

In this subsection, a short summary of COVID-19 is given, the data of which are sourced from the World Health Organization (https://covid19.who.int/, (accessed on 2 March 2022)) and Our World in Data (https://ourworldindata.org, (accessed on 2 March 2022)) [32]. These datasets cover the period from March 2020 to February 2022 on a daily basis. Figure 9 and Figure 10 give the mean values of new deaths and testing as per million/day, respectively. Figure 11 shows the statistical values, which are defined as follows:

$$\begin{array}{l} Values_{i}=Function\left(y_{i}\right),i\in \left[2020/3,2022/2\right]\\ y_{i}=mean\left(m-con_{ij}\right),j\in \{Africa,Asia,\cdots ,North\;America\}\\ m-con_{ij}=\max\left(x_{ij}^{l}\right),x_{ij}^{l}=\left\{\begin{array}{l} AdditionalAmountPer-million\\ AdditionalDeathAmountPer-million \end{array}\right. \end{array}$$

where $Function\left(\cdot \right)$ is the mean or standard deviation. Based on these figures, it is easy to observe that the epidemic very rapidly scaled up. Thus, building an intelligent process is necessary and helpful to advance medical services and protect the health of people.

**Figure 12:**
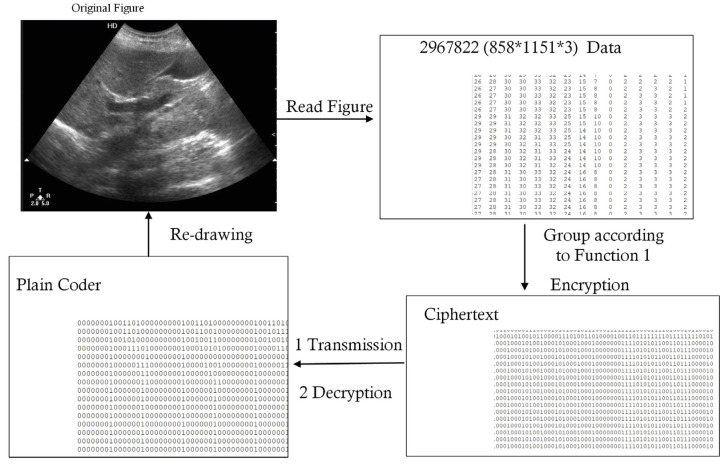
Flow of image.

In the setting of example case, two medical records need to be collected, one of which includes medical images; this case is used to simulate the process of transmission between hospitals and the OMC. The mechanism used to allocate Key-P is referred to in previous studies [24,33]. The patient first submits an application form, which includes the information derived from the medical record and the doctors taking part in the consultation, to the OMC. When the OMC accepts this application, the hospitals, which save a copy of the medical record, will send the ciphertext of the record, which is encrypted using a paired key (Key-P). Table 1 shows the text details of the medical record. The first record has 8379 characters, which includes 87 sensitive data items. DES is used to perform the task of symmetric encryption. Thus, the unit used in the process of encryption and transmission through the network is made up of 64 bits of binary digits. Combined with the code system designed in SMICTP (Methodology 2), eight characters that are present in one group need to be encrypted. Due to the space limitations, only one group is shown as an example in Table 1. Record 1 has 1037 groups, and Record 2 has 989 groups. It should be noted that if there are not enough characters to form a coding group, special characters are used to fill up the blank positions. Table 2 shows the information derived from the medical image listed in Record 2. According to SMICTP (Methodology 2), the medical image is first read via Image Acquisition into three basic color channels, with the plan digits having a size of 858*1151*3. Then, in order to distinguish them from character code, the Shifting Function for the figures defined in Function 1 is used in the raw image code. After shifting, each digit of the image needs 16; thus, each encryption group has four digits related to the image. For the example, there are 740,670 groups, which take 3433 s to be encrypted. Figure 12 illustrates the exact process of image handling. Besides the encryption process introduced above, the data are redrawn as an image after the encrypted binary digits arrive at the receiving end. 

### 4.3. Construction of Hash Value

In this work, SHA is set as the Hash function. Table 3 shows the results of different versions of SHA. It can be found that the lengths of the information abstract are not the same. The length of SHA_1 is 40 hexadecimals; however, the length of SHA3_384 is 96 hexadecimals, which is more than double that of SHA_1. Hash values are set as characters to reduce the complexity of encryption. And in order to unify the length to quickly package the medical data, the reserved length of the Hash values was 128 bytes, as defined in CSMP (Methodology 3). These bytes include tag bytes and the information abstract given by the SHA algorithm. The remainder is filled up with special values, which are shown in Table 4 and Table 5. Table 4 is the complete Hash value of the medical record from Hospital 1. For different versions of SHA, the length of padding is also shown. Table 5 shows the results of the medical record from Hospital 2. Then, the filled up values are encrypted using the same process as used for the letters using Key-P. The length of each Hash value is 128 bytes, each character of which can be expressed by 8 bits; thus, every Hash value needs to be partitioned into 16 groups. 

### 4.4. Validation 

Hash values and length checks are both employed to ensure the completeness of data. Firstly, the length check and Hash values are transmitted in the format of ciphertext; thus, they are not easy to modify. Secondly, the length check a provides quick and simple way to test whether modification was performed during the transmission. According to Methodology 3, three different lengths are used, of which Record 2 is listed in Table 6. Without the paired key, only the cryptograph can be read. Thus, it is not possible to modify the digits of lengths. After receiving the package, the lengths listed in the package are compared to the real length to quickly check whether the package is modified.

When the illegal modification of the package does not change the length, the quick check is invalid. The information abstract given by the Hash function can recognize this kind modification to ensure data completeness. The tables shown in the prior subsection show different versions of the Hash function, which involve the introduction of the tag in each version. Among the different versions, the encryption and employment of Hash values is the same; thus, only one value is set as an example in Table 7. The modification of data will be recognized by comparing the real value listed in the data package to the calculated value- after receiving package. Table 7 shows three ways to modify the medical data. These computational values are totally different from the real Hash value. Also, without the paired key, only ciphertext can be read, as shown in Table 8. Thus, it is not possible to modify the digits of Hash values and medical information at the same time.

Based on the prior analysis and verification of the proposed methodologies, it can be concluded that the scheme can provide a useful defense against the network attacks based on communication theory. In this work, a typical network attack known as a Man-in-the-Middle attack was used as an example to show this process. Man-in-the-Middle attacks play the role of a partner in the communication process. However, the total messages in our work are encrypted using symmetrical keys, which were defined in proposed methods. In particular, Methodology 3 showed these keys in an obvious way. In the proposed methods, symmetrical keys are only assigned by the OMC. Man-in-the-Middle attacks cannot obtain the encryption keys. Thus, any intercepted packaged in the networks can only be explained in a meaningless format. This approach has been verified in Table 1, Table 6, and Table 8. This approach is the only way to break the normal communication by cutting the package. However, three kinds of lengths in messages are used to provide a quick check about the completeness of data. Besides this observation, Hash values are useful in terms of ensuring the completeness of the data. Any change in the contents will generate totally different Hash values. In order to verify this point, three different modifications are used in Table 7. Another type of network attack, such as DDoS, makes the servers lose efficacy, since the services discussed in this work are not strong real-time tasks. At the same time, all works are based on computation. As a result, the mirror servers used as backups can decrease the damage caused by DDoS attacks.

## 5. Conclusions 

This work constructed a complete prototype of an online medical conference to provide consultation from doctors working in different hospitals. The proposed method balances the effectiveness and cost in terms of time and money by employing computer technology, which is useful in terms of building high-level communication protocols. Four methodologies were developed to ensure the secure transmission and authorization of medical information, namely SMED, SMICTP, CSMP, and SPMOC. SMICTP and SMED provide the complete framework of an online medical consultation conference, which allows the authorization of medical records and medical suggestion by employing communication and encryption technology. SMICTP describes medical record transmission, which uses a double-coding system to improve the recognition of transmission errors. CSMP and SPMOC mainly solve the problems that cannot be supported by the current technology. CSMP designs a special package for communication through public networks, which includes Hash values used to verify the accuracy of the data. For SPMOC, the Extended Chinese Remainder Theorem was first used to prove the effectiveness of solving the uncertain approval. Based on this approach, Channel-DT was proposed to complete the uncertain authorization, which is more suitable for real-world medical applications than current methods and technology. These works are all based on using the current level of software to achieve their goals. Thus, their operability and extension are improved compared to methods requiring a large number of hardware devices. 

## Figures and Tables

**Table 1:** Details of text information.

No	Original Length	Length of Non-Sensitive Data	Size of Each Group	Number of Groups	Example		
					Plain Language	Codes of Ciphertext	Ciphertext
1	8379	8292	8	1037	ccupatio	1110110011010110010111110001000101101101110111100001011000111001	ìÖ_\x11mÞ\x169
2	7979	7905	8	989	rrhea an	1010100011010010111010101111010100110010010111000000111101001000	¨Òêõ2\\\x0fH

**Table 2:** Details of image information.

Original Figure	Size of Plain Digits	Size of Each Group	Number of Groups	Encrypted Codes of One Group	Runtime
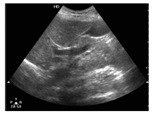	858*1151*3	4	740,670	1001100001111111001011001000111010101101000100011011011000010100	3433 s

**Table 3:** Information abstract of SHA.

No.	Kind	Standard	Length	Information Abstract
1	LS (Letter of Symbol)	sha1	40	4c6f3c5fcd56593e1bfe6729a48bb0f329f1ae63
		sha224	56	d943f15ff20d48a2c86cc23e40991248e20429151c22c5576b3836b5
		sha256	64	40041d5da76ec58f8e6dd2792faf5759e0021b8780132e10f9f30a8475e17821
		sha384	96	495c5bb90c873f617bfe4b75cd229c810b920612aa9fe285c54f7e34e78768cc1dc99967bcbac975319aec5d0321a149
		sha3_224	56	f65c2d1a4910b3569c2fe4a8f4d752f14b30b81a8a144496b18d8505
		sha3_256	64	8558a23afaaa4307bd26d6b5e7fb9b7bf4c507186510c758846c73208ac97a9a
		sha3_384	96	c6dbe8bbd77780bf60e6df2af117450b16b38cf34dc414aafd048f631a174b0b2fa829f3b70b2a31d207aed169f2da54
2	LS	sha1	40	a88a173108c6cf71667e38d183a1d9f3469e4366
		sha224	56	c42883342db9923da690d6fbe3ee4290e982e4490d3f2686ae970d34
		sha256	64	fcbb8592c47f709ea4a4a5620e40bb212b4ec1f9627c5fafcfcd6623e8f077af
		sha384	96	0b1c5d168e48821ced9488d5f38a6566dec58ec5f91675cd54b7524409cb56277de12b849eae1b48c9438c99ce8a5c74
		sha3_224	56	56055d1494a50197a84ba3a911c478904be72bcbb666e1df62553417
		sha3_256	64	07c175c7b8cbb0113626af152f2440d0d1d2376425394212dd2cfb20ac317587
		sha3_384	96	42a9d133600b3b986b7bf7beb5a50bacba27b087eaa3371bdb4c3bf97d179f65eb0d6a4e3c4b55c02974788f45129d6b
	F (Figures)	sha1	40	144d01528facc3a660b1887410e60f83a02d55f7
		sha224	56	955146e7c398ccd9050333df817971d3f8790f6d41710a0324ce16fb
		sha256	64	7f6edfd189195f8237cd0b622f397c5c79662457b8085017311ef5089be2f642
		sha384	96	c260d7d667c5dcf9d5aaa177aa8c5058f465a2edc4ef3fe342980090ec80123e505f05833a04b8bd8c82ea4a64e51552
		sha3_224	56	90e0bcf9896c55b253a73a5dd4bdd72376a8919adeab99c0dc607bf0
		sha3_256	64	d8737631fa5ad18a79a894fdb1c546f9c0f4d34be466732b4cf357a3f7b055f8
		sha3_384	96	d3bd7789420d320511db28562d8f488919d31c45fbb3027f59513027842916b334681aeed7277fac06c3232669d41b46

**Table 4:** Hash value of Record 1.

Standard	Tag	Filling	Plain Values
sha1	000	85	***000***00000000000000000000000000000000000000000000000000000000000000000000000000000000000004c6f3c5fcd56593e1bfe6729a48bb0f329f1ae63
sha224	001	69	***001***000000000000000000000000000000000000000000000000000000000000000000000d943f15ff20d48a2c86cc23e40991248e20429151c22c5576b3836b5
sha256	010	61	***010***000000000000000000000000000000000000000000000000000000000000040041d5da76ec58f8e6dd2792faf5759e0021b8780132e10f9f30a8475e17821
sha384	011	29	***011***00000000000000000000000000000495c5bb90c873f617bfe4b75cd229c810b920612aa9fe285c54f7e34e78768cc1dc99967bcbac975319aec5d0321a149
sha3_224	100	69	***100***000000000000000000000000000000000000000000000000000000000000000000000f65c2d1a4910b3569c2fe4a8f4d752f14b30b81a8a144496b18d8505
sha3_256	101	61	***101***00000000000000000000000000000000000000000000000000000000000008558a23afaaa4307bd26d6b5e7fb9b7bf4c507186510c758846c73208ac97a9a
sha3_384	110	29	***110***00000000000000000000000000000c6dbe8bbd77780bf60e6df2af117450b16b38cf34dc414aafd048f631a174b0b2fa829f3b70b2a31d207aed169f2da54

**Table 5:** Hash value of Record 2.

Kind	Standard	Tag	Filling	Plain Values
LS	sha1	000	85	***000***0000000000000000000000000000000000000000000000000000000000000000000000000000000000000a88a173108c6cf71667e38d183a1d9f3469e4366
	sha224	001	69	***001***000000000000000000000000000000000000000000000000000000000000000000000c42883342db9923da690d6fbe3ee4290e982e4490d3f2686ae970d34
	sha256	010	61	***010***0000000000000000000000000000000000000000000000000000000000000fcbb8592c47f709ea4a4a5620e40bb212b4ec1f9627c5fafcfcd6623e8f077af
	sha384	011	29	***011***000000000000000000000000000000b1c5d168e48821ced9488d5f38a6566dec58ec5f91675cd54b7524409cb56277de12b849eae1b48c9438c99ce8a5c74
	sha3_224	100	69	***100***00000000000000000000000000000000000000000000000000000000000000000000056055d1494a50197a84ba3a911c478904be72bcbb666e1df62553417
	sha3_256	101	61	***101***000000000000000000000000000000000000000000000000000000000000007c175c7b8cbb0113626af152f2440d0d1d2376425394212dd2cfb20ac317587
	sha3_384	110	29	***110***0000000000000000000000000000042a9d133600b3b986b7bf7beb5a50bacba27b087eaa3371bdb4c3bf97d179f65eb0d6a4e3c4b55c02974788f45129d6b
F	sha1	000	85	***000***0000000000000000000000000000000000000000000000000000000000000000000000000000000000000144d01528facc3a660b1887410e60f83a02d55f7
	sha224	001	69	***001***000000000000000000000000000000000000000000000000000000000000000000000955146e7c398ccd9050333df817971d3f8790f6d41710a0324ce16fb
	sha256	010	61	***010***00000000000000000000000000000000000000000000000000000000000007f6edfd189195f8237cd0b622f397c5c79662457b8085017311ef5089be2f642
	sha384	011	29	***011***00000000000000000000000000000c260d7d667c5dcf9d5aaa177aa8c5058f465a2edc4ef3fe342980090ec80123e505f05833a04b8bd8c82ea4a64e51552
	sha3_224	100	69	***100***00000000000000000000000000000000000000000000000000000000000000000000090e0bcf9896c55b253a73a5dd4bdd72376a8919adeab99c0dc607bf0
	sha3_256	101	61	***101***0000000000000000000000000000000000000000000000000000000000000d8737631fa5ad18a79a894fdb1c546f9c0f4d34be466732b4cf357a3f7b055f8
	sha3_384	110	29	***110***00000000000000000000000000000d3bd7789420d320511db28562d8f488919d31c45fbb3027f59513027842916b334681aeed7277fac06c3232669d41b46

**Table 6:** Encrypted length of Record 2.

Kind	Plain Language	Ciphertext
Total length	2-970-579	\x8buJÛ\r7óîÂ\x86ÕÆ\x9d]ZÃ
Character length	7905	\x8buJÛ\r7óîx«¸\xa0Ô\xad^¾
Extended length	2-970-835	\x8buJÛ\r7óîrÂ\x9c\x8aT¢Â0

**Table 7:** Comparison of Hash values.

Real Hash Value			Modification Way	Computational Value
Tag	Padding	Available Digits		
001	000000000000000000000000000000000000000000000000000000000000000000000	c42883342db9923da690d6fbe3ee4290e982e4490d3f2686ae970d34	Cover adjacent group	6d234b971ae211c3f0af11f6b15994005c11b0d161ec61b5fb951e11
			Change only one code	d8334a2bc746a0136ee18b5e2aab751656db5aeafd7f882eacc8e7fa
			Random digits—to be changed	b01d674c030fd1132130769fa75e8e9667e6993da84c9f1d6c9ea8d9

**Table 8:** Encrypted Hash values of Record 2.

Kind	Hash Value	Part of Encrypted Codes	Ciphertext
Text	001000000000000000000000000000000000000000000000000000000000000000000000c42883342db9923da690d6fbe3ee4290e982e4490d3f2686ae970d34	…0110010100011001010110101100010111111111100010100110000101010110…	Ï\x0by¸|E%~\x8buJÛ\r7óî\x8buJÛ\r7óî\x8buJÛ\r7óî\x8buJÛ\r7óî\x8buJÛ\r7óî\x8buJÛ\r7óî\x8buJÛ\r7óî\x8buJÛ\r7óîúòa\x0719[s\x91$îËo±\x97ae\x19ZÅÿ\x8aaV\xad:£Í¢\x1f\x13\x1fá«¿”ts\x0f8”§G@ú^2^/]Çû\x8f\x89^óû4
Figure	001000000000000000000000000000000000000000000000000000000000000000000000955146e7c398ccd9050333df817971d3f8790f6d41710a0324ce16fb	…0111101000010110111010110001111001111010010001100110001001001011…	Ï\x0by¸|E%~\x8buJÛ\r7óî\x8buJÛ\r7óî\x8buJÛ\r7óî\x8buJÛ\r7óî\x8buJÛ\r7óî\x8buJÛ\r7óî\x8buJÛ\r7óî\x8buJÛ\r7óîöõ\x8bÔ\x97,æ\x1fïs\x8a,Ô«õ\x92Æ\x13\x04\x9fXÏvSÚ\x01\x97\x95pö”;z\x16ë\x1ezFbKè¾\x01 = ø\x96Tï¶qa¾\xa0rÎG

## Data Availability

Part of the data used in this study were obtained from public datasets, the web address of which can be found in the main text; the other data are not available due to privacy concerns.

## References

[1] Nandy S., Adhikari M., Chakraborty S., Alkhayyat A., Kumar N. (2022). IBoNN: Intelligent Agent-based Internet of Medical Things framework for detecting brain response from Electroencephalography signal using Bag-of-Neural Network. Futur. Gener. Comput. Syst..

[2] Khan I.A., Moustafa N., Razzak I., Tanveer M., Pi D., Pan Y., Ali B.S. (2022). XSRU-IoMT: Explainable simple recurrent units for threat detection in Internet of Medical Things networks. Future Gener. Comput. Syst..

[3] Ghubaish A., Salman T., Zolanvari M., Unal D., Al-Ali A., Jain R. (2021). Recent Advances in the Internet-of-Medical-Things (IoMT) Systems Security. IEEE Internet Things J..

[4] Dimitrov D.V. (2016). Medical internet of things and big data in healthcare. Healthc. Inf. Res..

[5] Van der Velden B.H., Kuijf H.J., Gilhuijs K.G., Viergever M.A. (2022). Explainable artificial intelligence (XAI) in deep learning-based medical image analysis. Med. Image Anal..

[6] Zhao J., Hou X., Pan M., Zhang H. (2022). Attention-based generative adversarial network in medical imaging: A narrative review. Comput. Biol. Med..

[7] Bayılmış C., Ebleme M.A., Çavuşoğlu Ü., Küçük K., Sevin A. (2022). A survey on communication protocols and performance evaluations for Internet of Things. Digit. Commun. Netw..

[8] Qiao G., Liu Y., Zhou F., Zhao Y., Mazhar S., Yang G. (2022). Deep learning-based M-ary spread spectrum communication system in shallow water acoustic channel. Appl. Acoust..

[9] Wang Y., Liu Y., Mu J., Feng Z., Tang X. (2021). Collimating/focusing optical system designed for hard X-ray communication. Nucl. Instrum. Methods Phys. Res. Sect. A Accel. Spectrometers Detect. Assoc. Equip..

[10] Liu C., Liu S., Yang Y., Zhao K., Chen Y., Su Y., Zhang K., Feng J. (2022). The FFIS spectrometer for determination of fission fragment mass distribution with the energy–velocity method. Nucl. Instrum. Methods Phys. Res. Sect. A Accel. Spectrometers Detect. Assoc. Equip..

[11] Eklöf B., Larsson H., Ellbin S., Jonsdottir I.H., O’Dwyer S., Hansson C. (2022). The role of self-reported stressors in recovery from Exhaustion Disorder: A longitudinal study. BMC Psychiatry.

[12] Li X., Laplante D.P., Paquin V., Lafortune S., Elgbeili G., King S. (2022). Effectiveness of cognitive behavioral therapy for perinatal maternal depression, anxiety and stress: A systematic review and meta-analysis of randomized controlled trials. Clin. Psychol. Rev..

[13] Tsai Z., Kiss A., Nadeem S., Sidhom K., Owais S., Faltyn M., Van Lieshout R.J. (2022). Evaluating the effectiveness and quality of mobile applications for perinatal depression and anxiety: A systematic review and meta-analysis. J. Affect. Disord..

[14] Elmisery A.M., Rho S., Aborizka M. (2019). A new computing environment for collective privacy protection from constrained healthcare devices to IoT cloud services. Clust. Comput..

[15] Wu K., Tian B., Wang X., Zhao Q. A Secure and Anonymous Communication Scheme Based on Internet Public Service Environment. Proceedings of the 2022 International Conference on Networking and Network Applications (NaNA).

[16] Dharminder D., Mishra D., Li X. (2020). Construction of RSA-based authentication scheme in authorized access to healthcare services. J. Med. Syst..

[17] Shreya S., Chatterjee K., Singh A. (2022). A smart secure healthcare monitoring system with Internet of Medical Things. Comput. Electr. Eng. Vol..

[18] Renuka K., Kumari S., Li X. (2019). Design of a secure three-factor authentication scheme for smart healthcare. J. Med. Syst..

[19] Zhao X., Xiao W., Wu L., Zhao Z., Huo J., Wang S., Guo Z., Sun D. (2020). Intelligent city intelligent medical sharing technology based on internet of things technology. Future Gener Comput. Syst..

[20] He Y., Camacho R.S., Soygazi H., Luo C. (2021). Attacking and defence pathways for Intelligent Medical Diagnosis System (IMDS). Int. J. Med Inform..

[21] Yaqoob T., Abbas H., Atiquzzaman M. (2019). Security vulnerabilities, attacks, countermeasures, and regulations of networked medical devices—A review. IEEE Commun. Surv. Tutor..

[22] Soni M., Singh D.K. (2022). Privacy-preserving secure and low-cost medical data communication scheme for smart healthcare. Comput. Commun..

[23] Mishra N., Islam S.H., Zeadally S. (2021). A comprehensive review on collision-resistant hash functions on lattices. J. Inf. Secur. Appl..

[24] Stinson D. (2002). Cryptography: Theory and Practice.

[25] Choi M., Lee J., Kim S., Jeong Y.-S., Park J.-H. (2016). Location based authentication scheme using BLE for high performance digital content management system. Neurocomputing.

[26] Ullah F., Pun C.-M. (2023). Deep self-learning based dynamic secret key generation for novel secure and efficient hashing algorithm. Inf. Sci..

[27] Pfitzmann A., Aβmann R. (1993). More efficient software implementations of (generalized) DES. Comput. Secur..

[28] Kamphenkel K., Blank M., Bauer J., Carle G. Adaptive Encryption for the Realization of Real-Time Transmission of Sensitive Medical Video Streams. Proceedings of the 2008 International Symposium on a World of Wireless, Mobile and Multimedia Networks.

[29] Vida M., Gomoi V., Tivadar L.S., Stoicu-Tivadar V. Generating Medical Computer-Based Protocols Using Standardized Data Transmission. Proceedings of the 4th International Workshop on Soft Computing Applications.

[30] Epel B., Halpern H.J. (2021). EPR Oxygen Imaging Workflow with MATLAB Image Registration Toolbox. Appl. Magn. Reson..

[31] Ding C., Pei D., Salomaa A. (1996). Chinese Remainder Theorem. Applications in Computing, Coding, Cryptography.

[32] Hasell J., Mathieu E., Beltekian D., Macdonald B., Giattino C., Ortiz-Ospina E., Roser M., Ritchie H. (2020). A cross-country database of COVID-19 testing. Sci. Data.

[33] Setyaningsih E., Wardoyo R., Sari A.K. (2020). Securing color image transmission using compression-encryption model with dynamic key generator and efficient symmetric key distribution. Digit. Commun. Netw..

